# Genome-wide identification and expression profiling of *trihelix* gene family under abiotic stresses in wheat

**DOI:** 10.1186/s12864-019-5632-2

**Published:** 2019-04-11

**Authors:** Jie Xiao, Rui Hu, Ting Gu, Jiapeng Han, Ding Qiu, Peipei Su, Jialu Feng, Junli Chang, Guangxiao Yang, Guangyuan He

**Affiliations:** The Genetic Engineering International Cooperation Base of Chinese Ministry of Science and Technology, Key Laboratory of Molecular Biophysics of Chinese Ministry of Education, College of Life Science and Technology, Huazhong University of Science and Technology (HUST), Wuhan, 430074 China

**Keywords:** Wheat, *trihelix* gene family, Transcription factor, Orthology relation, Expression profile, Abiotic stress

## Abstract

**Background:**

The *trihelix* gene family is a plant-specific transcription factor family that plays important roles in plant growth, development, and responses to abiotic stresses. However, to date, no systemic characterization of the *trihelix* genes has yet been conducted in wheat and its close relatives.

**Results:**

We identified a total of 94 *trihelix* genes in wheat, as well as 22 *trihelix* genes in *Triticum urartu*, 29 in *Aegilops tauschii*, and 31 in *Brachypodium distachyon*. We analyzed the chromosomal locations and orthology relations of the identified *trihelix* genes, and no *trihelix* gene was found to be located on chromosome 7A, 7B, or 7D of wheat, thereby reflecting the uneven distributions of wheat *trihelix* genes. Phylogenetic analysis indicated that the 186 identified trihelix proteins in wheat, rice, *B. distachyon*, and *Arabidopsis* were clustered into five major clades. The *trihelix* genes belonging to the same clades usually shared similar motif compositions and exon/intron structural patterns. Five pairs of tandem duplication genes and three pairs of segmental duplication genes were identified in the wheat *trihelix* gene family, thereby validating the supposition that more intrachromosomal gene duplication events occur in the genome of wheat than in that of other grass species. The tissue-specific expression and differential expression profiling of the identified genes under cold and drought stresses were analyzed by using RNA-seq data. qRT-PCR was also used to confirm the expression profiles of ten selected wheat *trihelix* genes under multiple abiotic stresses, and we found that these genes mainly responded to salt and cold stresses.

**Conclusions:**

In this study, we identified *trihelix* genes in wheat and its close relatives and found that gene duplication events are the main driving force for *trihelix* gene evolution in wheat. Our expression profiling analysis demonstrated that wheat *trihelix* genes responded to multiple abiotic stresses, especially salt and cold stresses. The results of our study built a basis for further investigation of the functions of wheat *trihelix* genes and provided candidate genes for stress-resistant wheat breeding programs.

**Electronic supplementary material:**

The online version of this article (10.1186/s12864-019-5632-2) contains supplementary material, which is available to authorized users.

## Background

Transcription factors (TFs) are extensively involved in the processes of plant growth and development through binding to specific *cis*-elements to modulate the expressions of target genes [1]. More than 60 TF families have been found in plants, many of which have been thoroughly studied to evaluate their functions in grasses [2]. As one of the first TFs discovered in plants, trihelix proteins widely participate in diverse development processes and abiotic stress responses [3]. Trihelix TFs are plant-specific, suggesting that they might be involved in plant-specific gene regulations [4]. However, the *trihelix* gene family has not been systematically identified in wheat or its closely related plant species. Trihelix TFs contain one or two trihelix DNA-binding domains which could specifically bind to the GT elements of promoters. The trihelix DNA-binding domain contains a typical trihelix structure (three α-helixes separated by two loops) and is similar to the Myb DNA-binding domains in sequence [5].

The first identified *trihelix* gene *GT-1* was found in *Pisum sativum*, and orthologous genes were subsequently cloned in tobacco and *Arabidopsis* [6–9]. GT-1 protein directly interacts with pre-initiation complex and activates transcription. Early studies on *Arabidopsis* suggest that *trihelix* genes play multiple roles in diverse development processes. *Arabidopsis* ASIL1 targets GT-box-containing embryonic genes and represses the expression of embryonic seed maturation genes in vegetative tissues [10, 11]. The gain-of-function *Arabidopsis* mutant of the *PTL* (*PETAL LOSS*) gene causes male sterility and other pleiotropic phenotypes [12]. GTL1 has been reported to be involved in regulating ploidy-dependent cell growth in the *Arabidopsis* trichome [13].

In recent years, evidence has shown that trihelix proteins are extensively involved in the plant response to different abiotic stresses. Overexpression of *GmGT-2A* and *GmGT-2B* in *Arabidopsis* could improve its tolerance to abiotic stresses [14]. The *OsGTγ-1* gene has also been proven to respond to salt stress in rice [15]. GTL1 affects plant water use efficiency and its tolerance to drought stress [16]. AtGT2L could interact with calmodulin and is involved in the abiotic stress response [17]. AtGT-4 modulates *Arabidopsis* salt tolerance by interacting with TEM2 [18]. TaGT2L1D regulates plant development and drought tolerance in wheat [19]. ShCIGT regulates the cold and drought tolerance of tomato by interacting with SnRK1 [20]. By binding to AGAG-Box, AtAST1 mediates *Arabidopsis* salt and osmotic stress tolerance [21].

A total of 30 and 31 trihelix proteins have been identified in *Arabidopsis* and rice, respectively, and these proteins could be divided into five clades named GT-1, GT-2, GTγ, SH4, and SIP1 [4, 11]. Trihelix proteins were subsequently identified in many other plant species. Prior studies report 63 *trihelix* genes in *Glycine max*, 36 in *Solanum lycopersicum*, 20 in *Chrysanthemum morifolium*, 56 in *Populus trichocarpa*, 10 in *Camellia sinensis*, and 52 in *Brassica rapa* [22–27].

As an important cereal crop, wheat has a very high yield. According to the latest forecast from the Food and Agriculture Organization of the United Nations (FAO, http://www.fao.org/worldfoodsituation/csdb/en/), global cereal production in 2018 was 2.59 billion tonnes, of which 722.4 million tonnes was wheat yield. Crop productivity and food security are affected by many factors, including diverse environmental factors, such as drought, salt, and temperature stresses. As global climate change intensifies, the adverse effects of these abiotic stresses may be enhanced [28, 29]. Studies on wheat *trihelix* genes could contribute to stress-resistant crop breeding.

In the present study, the *trihelix* gene family was identified in hexaploid bread wheat (*Triticum aestivum* L.) and its relatives, including *Triticum urartu*, *Aegilops tauschii*, and *Brachypodium distachyon*. The chromosomal distributions, protein characteristics, gene structures, and conserved motif compositions of the identified *trihelix* genes were analyzed. We then identified orthology relations, analyzed gene duplication events, and constructed the phylogenetic trees of the identified *trihelix* genes. Using public RNA-seq data, we analyzed the spatial and temporal expressions and differential expression profiles of wheat *trihelix* genes under abiotic stresses. In addition, our qRT-PCR results validated the supposition that wheat *trihelix* genes participate in various abiotic stress responses. Our research provides valuable clues for future functional characterization of *trihelix* genes in wheat.

## Results

### Identification of *trihelix* genes

The Hidden Markov Model (HMM) profile of trihelix domain (PF13837) was used to search the trihelix domain in the wheat protein database (IWGSC RefSeq v1.0). The SMART and HMMER websites were used to confirm that all candidate genes identified contain the trihelix domain. A total of 94 non-redundant *trihelix* genes were identified in wheat (Additional file 1: Tables S1–S3). To confirm the reliability of the above identification, 31 published rice *trihelix* genes were used to search their homologous genes in wheat on NCBI by BLAST, and we found that all published wheat proteins containing the trihelix domain were included. The wheat *trihelix* genes were named from *TaGT-1* to *TaGT-94* based on their positions on the chromosomes. Additionally, *TaGT2L1A*, *TaGT2L1B,* and *TaGT2L1D* have been identified and named in previous studies [19], and these genes were found to be identical to *TaGT-10*, *TaGT-41*, and *TaGT-73*, respectively, in our 94 wheat *trihelix* genes.

*T. urartu* (diploid, AA) and *Ae. tauschii* (diploid, DD) are the progenitors of the hexaploid wheat (*T. aestivum*, AABBDD). The wild grass *B. distachyon* is the first sequenced member of the Pooideae subfamily. These grasses have a close genetic relationship with wheat. We identified 22, 29, and 31 *trihelix* genes in *T. urartu*, *Ae. tauschii*, and *B. distachyon*, respectively (Additional file 1: Tables S1–S3).

We predicted the isoelectric point (pI) and molecular weight (Mw) of *trihelix* genes by using the ExPASy pI/Mw tool. Wheat trihelix proteins were found to have large variations in length in the range of 197–851 amino acid residues. The trihelix proteins in wheat also varied greatly in pI (5.14–10.57) and Mw (21.6–94.4 kDa), and they showed similar variations in *T. urartu*, *Ae. tauschii*, and *B. distachyon* (Additional file 1: Table S1). Given that subcellular localization information could provide certain clues for the protein function study, subcellular localizations of trihelix proteins in wheat were predicted by using WOLF PSORT (Additional file 1: Table S1). The results of subcellular localization prediction of wheat trihelix proteins showed that most trihelix proteins are located in the nuclei, in accordance with their roles as TFs.

### Chromosomal distribution of *trihelix* genes

**Fig. 1:**
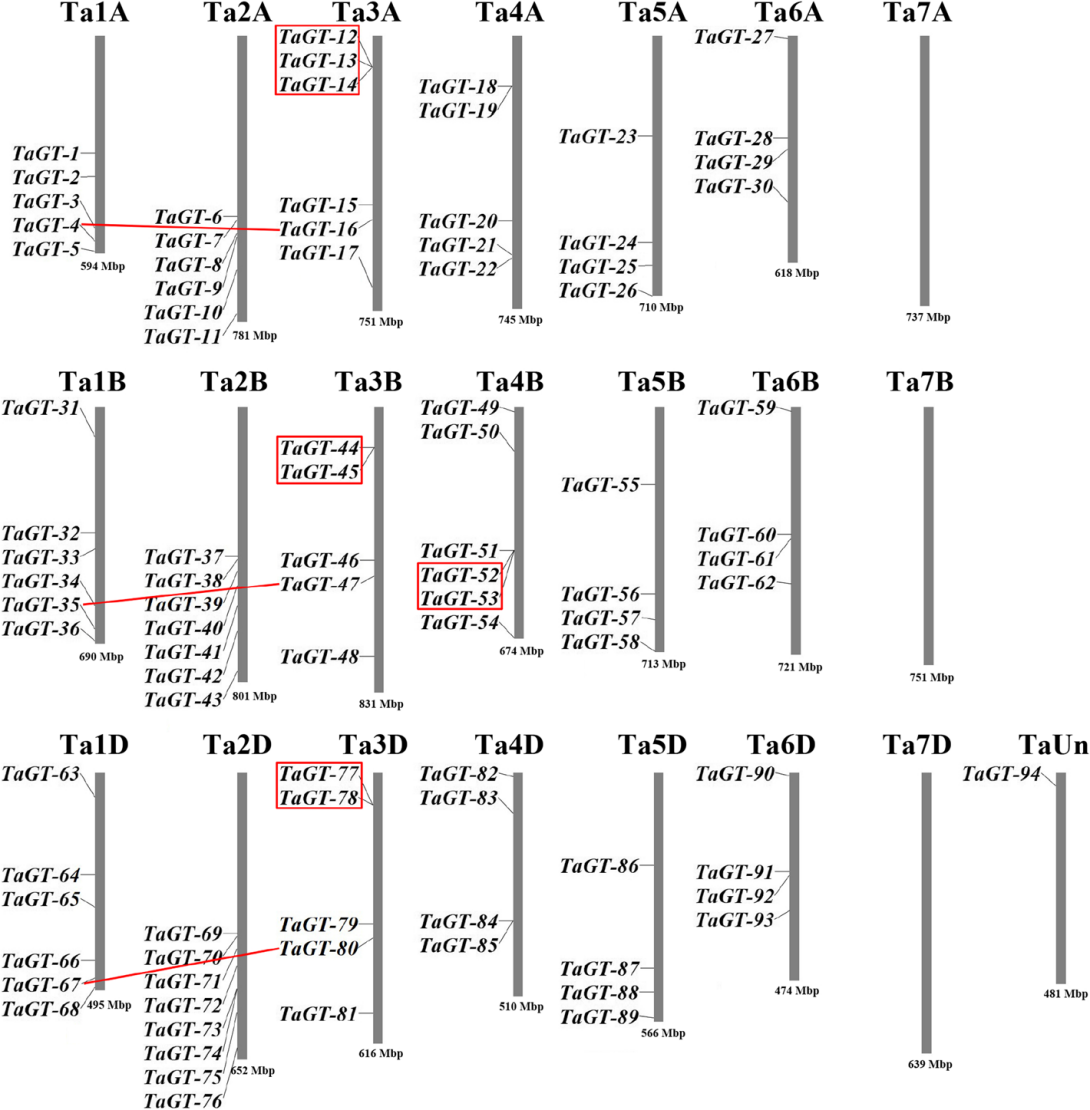
Locations and duplication events of *trihelix* genes on wheat chromosomes. Red boxes indicate tandem duplications, and red lines indicate segmental duplications. The image was drawn via MapInspect

The positions of *trihelix* genes were obtained from the genome annotation files. A total of 30, 32, and 31 wheat *trihelix* genes were located on subgenomes A, B, and D (Fig. 1, Additional file 1: Table S1), respectively. *TaGT-94* was located on chromosome TaUn because of the incomplete wheat genome sequence. Given that *TaGT-26* and TaGT*-54* are homologous with *TaGT-94*, we speculated that the actual location of *TaGT-94* might be located on the distal of chromosome 4DL or 5DL. Chromosome 2D had eight genes, the largest number of wheat *trihelix* genes found in a single chromosome. No *trihelix* gene was located on chromosome 7A, 7B, or 7D. The numbers of *trihelix* genes distributed in the remaining chromosomes showed little difference, and relatively high densities were detected at the distal of each chromosome.

The *trihelix* genes in *T. urartu*, *Ae. tauschii*, and *B. distachyon* were named according to their positions and tended to distribute at the distal of each chromosome (Additional file 2: Figures S1–S3, Additional file 1: Table S1). No *trihelix* gene was located on chromosome 7A of *T. urartu* or 7D of *Ae. tauschii*. *Trihelix* genes existed on all 5 chromosomes of *B. distachyon* and 11 genes located on chromosome Bd3, which showed the largest number of *trihelix* genes.

### Identification of orthologs between wheat and its relatives

Given that orthology relations reflect species phylogenies and can be used to transfer annotations from a known gene to another newly-sequenced genome, ortholog identification has become very important [30, 31]. Here, we used OrthoGNC software to predict pairwise orthologous genes among wheat, *T. urartu*, *Ae. tauschii*, *B. distachyon*, and rice (Table 1).

**Table 1:** Orthologs of *trihelix* genes among wheat and its relatives

Wheat Subgenome A	*T. urartu* (AA)	Wheat Subgenome B	Wheat Subgenome D	*Ae. tauschii* (DD)	*B. distachyon*	Rice
	*TuGT-1*	*TaGT-31*	*TaGT-63*	*AetGT-1*	*BdGT-6*	*Os05g03740.1*
*TaGT-1*	*TuGT-2*	*TaGT-32*	*TaGT-64*	*AetGT-2*	*BdGT-13*	*Os10g37240.2*
*TaGT-2*	*TuGT-3*	*TaGT-33*	*TaGT-65*	*AetGT-3*	*BdGT-14*	*Os10g41460.1*
	*TuGT-4*					
*TaGT-3*		*TaGT-34*	*TaGT-66*	*AetGT-4*	*BdGT-2*	*Os03g18330.1*
*TaGT-4*		*TaGT-35*	*TaGT-67*	*AetGT-5*	*BdGT-5*	*Os05g48690.1*
*TaGT-5*	*TuGT-5*	*TaGT-36*	*TaGT-68*	*AetGT-6*	*BdGT-21*	*Os11g06410.1*
*TaGT-6*	*TuGT-6*	*TaGT-37*	*TaGT-69*	*AetGT-7*	*BdGT-24*	*Os04g32590.1*
*TaGT-7*	*TuGT-7*	*TaGT-38*	*TaGT-70*		*BdGT-25*	*Os04g33300.1*
*TaGT-8*		*TaGT-39*	*TaGT-71*	*AetGT-8*	*BdGT-26*	*Os04g36790.1*
*TaGT-9*	*TuGT-8*	*TaGT-40*	*TaGT-72*	*AetGT-9*	*BdGT-27*	*Os04g40930.1*
*TaGT-10*	*TuGT-9*	*TaGT-41*	*TaGT-73*	*AetGT-10*	*BdGT-28*	*Os04g45750.1*
			*TaGT-74*	*AetGT-11*	*BdGT-29*	*Os04g45940.1*
	*TuGT-10*	*TaGT-42*	*TaGT-75*		*BdGT-30*	*Os04g51320.1*
*TaGT-11*	*TuGT-11*	*TaGT-43*	*TaGT-76*	*AetGT-12*	*BdGT-31*	*Os04g57530.1*
*TaGT-12*	*TuGT-12*	*TaGT-44*	*TaGT-77*	*AetGT-13*	*BdGT-4*	*Os01g21590.1*
*TaGT-13*		*TaGT-45*	*TaGT-78*	*AetGT-14*		
*TaGT-14*	*TuGT-13*					
*TaGT-15*	*TuGT-14*	*TaGT-46*	*TaGT-79*	*AetGT-15*	*BdGT-8*	*Os01g52090.1*
*TaGT-16*	*TuGT-15*	*TaGT-47*	*TaGT-80*	*AetGT-16*	*BdGT-7*	*Os01g48320.1*
*TaGT-17*		*TaGT-48*	*TaGT-81*	*AetGT-17*	*BdGT-9*	*Os01g70230.1*
		*TaGT-53*				
*TaGT-18*	*TuGT-17*	*TaGT-52*	*TaGT-85*	*AetGT-21*		
*TaGT-19*	*TuGT-18*	*TaGT-51*	*TaGT-84*	*AetGT-20*		*Os03g18340.1*
*TaGT-20*		*TaGT-50*	*TaGT-83*	*AetGT-19*		
*TaGT-21*	*TuGT-16*	*TaGT-49*	*TaGT-82*	*AetGT-18*	*BdGT-1*	*Os03g46350.1*
*TaGT-22*		*TaGT-58*	*TaGT-89*		*BdGT-15*	*Os08g37810.1*
*TaGT-23*	*TuGT-19*	*TaGT-55*	*TaGT-86*	*AetGT-23*	*BdGT-23*	*Os12g06640.1*
*TaGT-24*		*TaGT-56*	*TaGT-87*	*AetGT-24*	*BdGT-22*	*Os09g38570.1*
*TaGT-25*		*TaGT-57*	*TaGT-88*	*AetGT-25*	*BdGT-16*	*Os02g31160.1*
*TaGT-26*		*TaGT-54*	*TaGT-94*	*AetGT-22*	*BdGT-3*	*Os03g02240.1*
*TaGT-27*		*TaGT-59*	*TaGT-90*	*AetGT-26*	*BdGT-10*	*Os02g01380.1*
*TaGT-28*	*TuGT-21*	*TaGT-61*	*TaGT-91*	*AetGT-28*	*BdGT-17*	*Os02g33610.1*
*TaGT-29*		*TaGT-60*	*TaGT-92*	*AetGT-27*	*BdGT-19*	*Os02g35690.1*
*TaGT-30*	*TuGT-22*	*TaGT-62*	*TaGT-93*	*AetGT-29*	*BdGT-20*	*Os02g43300.1*
	*TuGT-20*				*BdGT-11*	*Os02g07800.1*
					*BdGT-12*	
					*BdGT-18*	*Os02g33770.1*

Wheat is an allohexaploid species with a complex genetic background derived from two naturally interspecific hybridization events of three diploid donor species [32, 33]. Therefore, each wheat gene usually has three homologous copies. Using BLASTP, orthologous relationships among all wheat *trihelix* genes were identified and are described in Table 1. Likely due to gene loss or the incomplete genome sequence, no gene was identified on subgenome A as a homologous gene with *TaGT-31* and *TaGT-63*, and no gene was identified on subgenome A or B as homologous with *TaGT-74*. Of particular interest here is that *TaGT-42* and *TaGT-75* had a homologous gene *TraesCS2A02G436000.1* located on subgenome A, which reveals fragment deletion of 170 amino acids at the N-terminal containing a trihelix domain; by contrast, nearly no amino acid difference was found at the C-terminal. Based on this finding, *TraesCS2A02G436000.1* was not identified as a *trihelix* gene in our study.

### Phylogenetic analysis and genome synteny analysis of *trihelix* genes

**Fig. 2:**
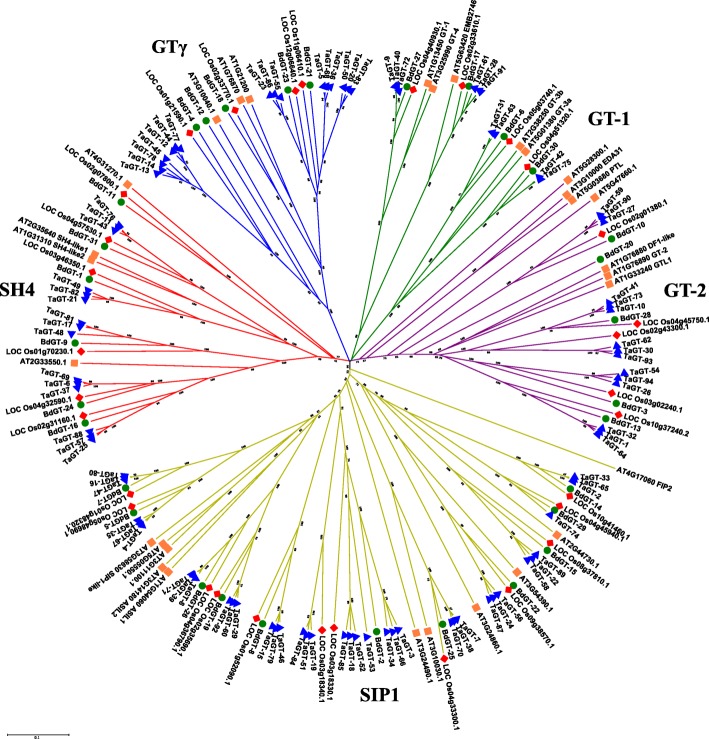
Phylogenetic tree of trihelix proteins in wheat, *B. distachyon*, rice, and *Arabidopsis*. The unrooted tree was drawn by MEGA 7.0 with the sequences of 186 identified trihelix proteins. The bootstrap value was set to 1000 replicates

Using MEGA7, we constructed an unrooted phylogenetic tree following the full amino acid sequences of 186 identified trihelix proteins in *Arabidopsis*, rice, *B. distachyon*, and wheat (Fig. 2). Trihelix proteins were clustered into five major clades, consistent with the results in *B. rapa* and *P. trichocarpa* [22, 24]. The five major clades were named GT-1, GT-2, SIP1, SH4, and GTγ based on studies on rice and *Arabidopsis* [4], and these clades respectively contained 10, 15, 38, 15, and 16 wheat trihelix proteins (Additional file 1: Table S1). The distribution trends were similar to those in *Arabidopsis* and rice: the SIP1 clade contained the maximum number of members and the GT-1 clade contained the minimum number of members.

Both tandem and segmental duplications are essential to gene family evolution for adapting to various environmental conditions [34, 35]. In this study, five pairs of genes among 94 *trihelix* genes of wheat were identified as tandem duplications, and three pairs of genes were considered segmental duplications (Fig. 1). *TuGT-12*/*TuGT-13* was the only pair of tandem duplication genes identified in *T. urartu* (AA) (Additional file 2: Figure S1), and these genes were orthologous with *TaGT-13*/*TaGT-14* in wheat subgenome A. *AetGT-13*/*AetGT-14* were the only pair of tandem duplication genes identified in *Ae. tauschii* (DD) (Additional file 2: Figure S2), and these genes were orthologous with *TaGT-77/TaGT-78* in wheat subgenome D. *TaGT-13*/*TaGT-14* were homologous with *TaGT-77*/*TaGT-78*. These results suggest that the tandem duplication genes *TaGT-13*/*TaGT-14* formed before the interspecific hybridization events of three diploid donor species. During the speciation and evolution of bread wheat, a new duplication event occurred, and the tandem duplication genes *TaGT-12*/*TaGT-13* were generated. No segmental duplication gene was identified in *T. urartu*. We identified *AetGT-5*/*AetGT16* as a pair of segmental duplication genes in *Ae. tauschii*. No *trihelix* gene in *B. distachyon* was identified as a tandem duplication, and 10 pairs of genes were found to be segmental duplications (Additional file 2: Figure S3).

To analyze the synteny relationships of *trihelix* genes between *T. aestivum*, *B. distachyon*, and rice, we used the Multiple Collinearity Scan toolkit (MCScanX). Approximately 80.6% (25 of 31) the rice *trihelix* genes exhibited synteny with *trihelix* genes in wheat (Additional file 2: Figure S4, Additional file 1: Table S4). Furthermore, 74.2% (23 of 31) of the *trihelix* genes in *B. distachyon* were found to have synteny with wheat *trihelix* genes (Additional file 2: Figure S5, Additional file 1: Table S4).

### Motif composition and gene structure analysis of the *trihelix* genes

The conserved motifs of *trihelix* genes in *Arabidopsis*, rice, *B. distachyon*, and wheat were analyzed by using MEME Suite tool. Motif logos were also obtained (Additional file 2: Figure S6). A total of 13 conserved motifs were identified. Gene Structure Display Server 2.0 was used to analyze and visualize the exon and intron structures of *trihelix* genes in wheat and *B. distachyon*.

**Fig. 3:**
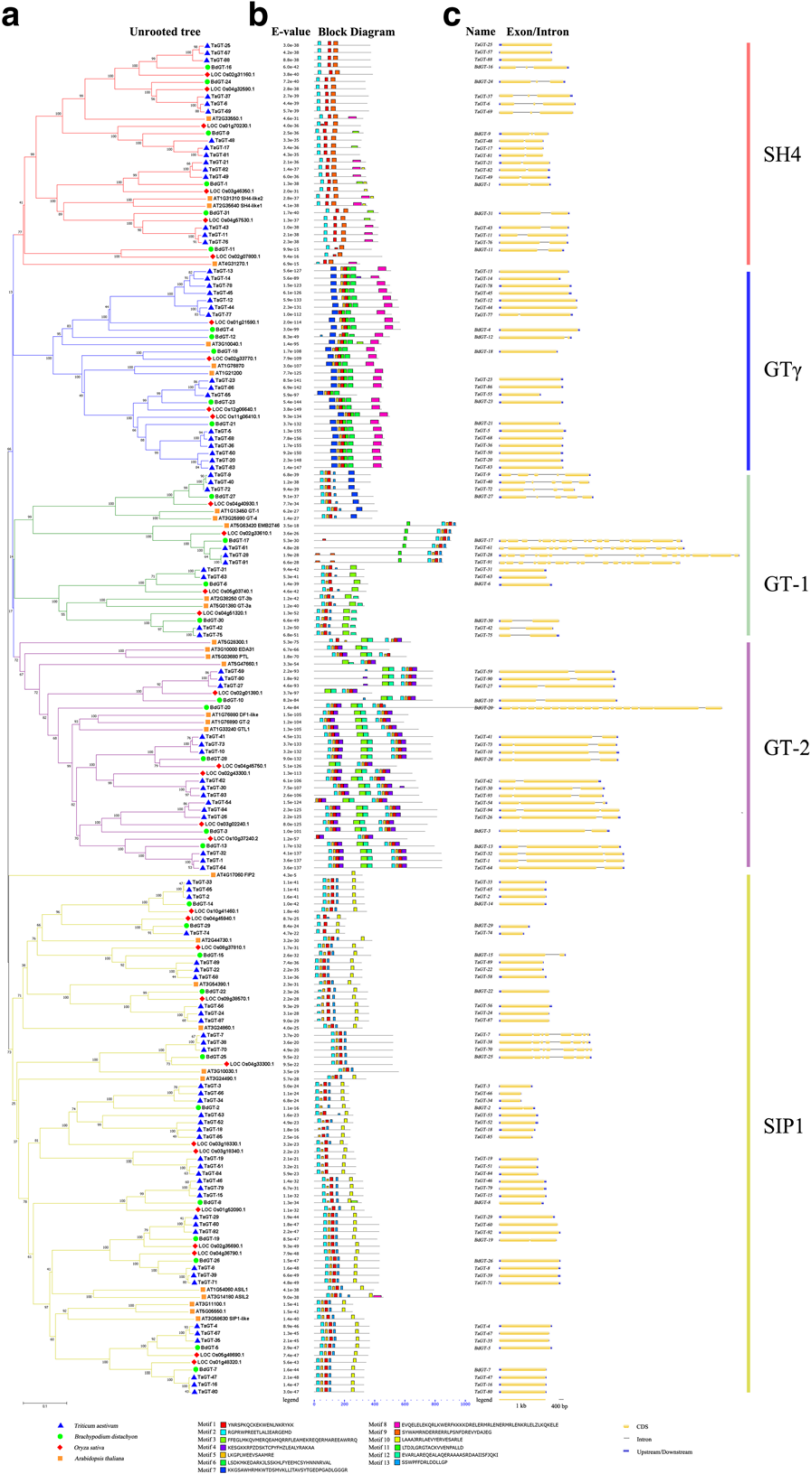
Phylogenetic tree, motif compositions, and gene structures of *trihelix* genes. **a** Unrooted neighbor-joining phylogenetic tree. The tree was built using trihelix protein sequences of *Arabidopsis*, rice, *B*. *distachyon,* and wheat. **b** Schematic representation of conserved motifs. Colored boxes indicate different conserved motifs. **c** Exon/intron organization. Exons are shown as yellow boxes, and introns are shown as gray lines

*Trihelix* genes belonging to the same clade usually have similar motif compositions and exon/intron structures (Fig. 3), thereby indicating that they may have similar functions. Except for GTγ clade genes, all trihelix proteins contained motif 1 and motif 2. GTγ clade genes and some SH4 clade genes featured motif 8 at their C-terminal, and all SIP1 clade genes contained motif 10 at their C-terminal. GT-2 clade genes had two trihelix domains, with motif 3 and motif 12 in the middle of the protein sequence. The majority of *trihelix* genes (90% in wheat, 87% in *B. distachyon*) had very few introns (0–2). All GTγ clade genes had no intron (one exon). Some members belonging to GT-1 and GT-2 clades had more than 10 introns. *TaGT-28*, *TaGT-61*, and *TaGT-91* are homologs and the longest *trihelix* genes in wheat, containing 16 introns. In *B. distachyon*, *BdGT-20* and *BdGT-17* are the longest *trihelix* genes, containing 19 and 16 introns, respectively.

### Tissue-specific expression analysis of *trihelix* genes in wheat

**Fig. 4:**
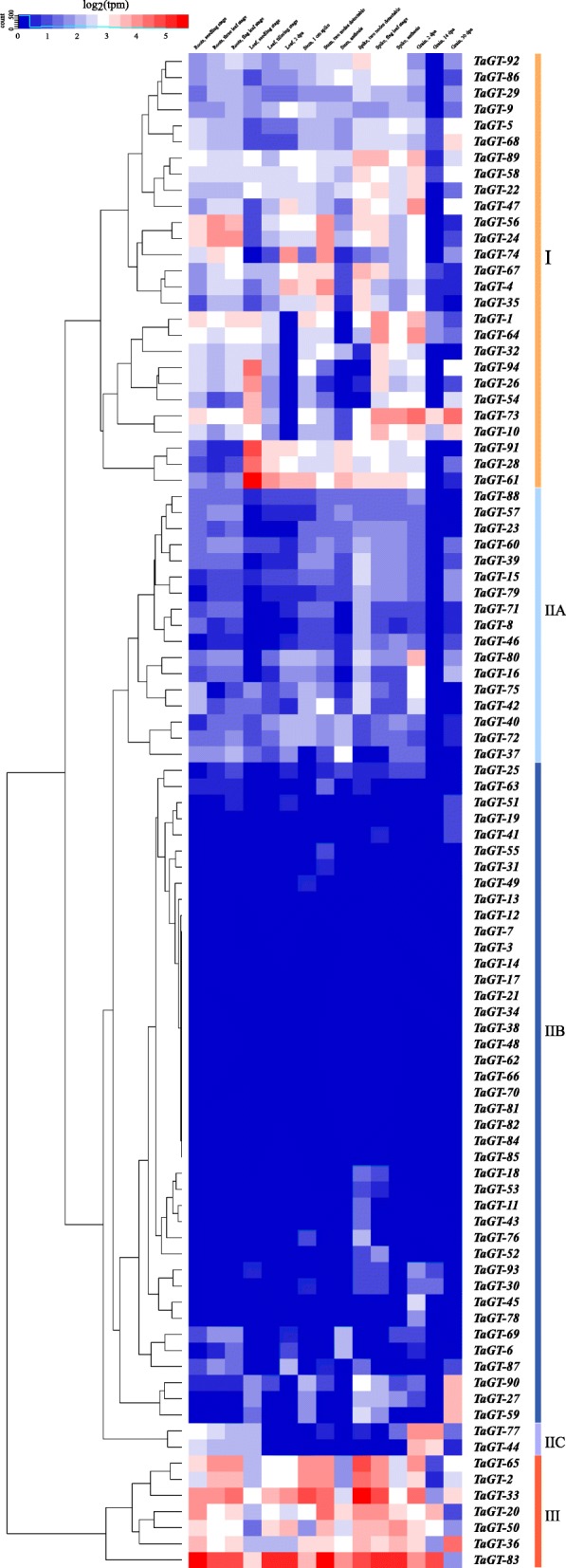
Tissue-specific expression profiles of wheat *trihelix* genes. The heatmap was drawn using R. The color scale indicates the log2 values of transcript per million. The colors red and blue represent higher and lower expression levels, respectively

To analyze the tissue-specific expression profiles of 94 wheat *trihelix* genes, public RNA-seq data of wheat cv. Chinese Spring was obtained from the expVIP website. The data covered gene expression profiles of different tissues including root, stem, leaf, spike, and grain throughout the entire life cycle of wheat. Hierarchical cluster analysis was conducted based on the log2 of transcript per million (TPM) values of 94 wheat *trihelix* genes (Fig. 4, Additional file 1: Table S5).

The tissue expression profiles of *trihelix* genes in wheat were divided into three groups based on their expression characteristics. Group I contained 27 genes, and their average expression levels in TPM ranged from 3.47 to 10.14 (average value = 5.82). The expression levels of these genes showed remarkable differences at different stages in different tissues. Group II included 60 genes that had relatively low expression (average value = 1.23) in most tissues, and this group could be divided into three subgroups. Subgroup IIA comprised 17 genes with tissue-specific expressions. Subgroup IIB consisted of 41 genes with nearly negligible expression in all the tissues. Subgroup IIC only included 2 genes, which were specifically highly expressed in grain. Group III was composed of 7 genes, and these genes were highly expressed in almost all the tissues (average value = 13.63).

The wheat *trihelix* genes belonging to the same clade did not have the same expression profiles. However, three homologous copies often had similar tissue expression profiles. For example, *TaGT-26*, *TaGT-54*, and *TaGT-94* are homologous genes belonging to group I, and they were all highly expressed in seedling leaves and lowly expressed in roots and grain. Homologous genes *TaGT-21*, *TaGT-49*, and *TaGT-82*, which belong to group II, were barely expressed in nearly all tissues. Homologous genes *TaGT-20*, *TaGT-50*, and *TaGT-83*, which belong to group III, had relatively high transcript levels in the majority of tissues. Among the *trihelix* genes in wheat, *TaGT-83* had the highest average transcript level.

### Expression profiling analysis of *trihelix* genes in wheat

**Fig. 5:**
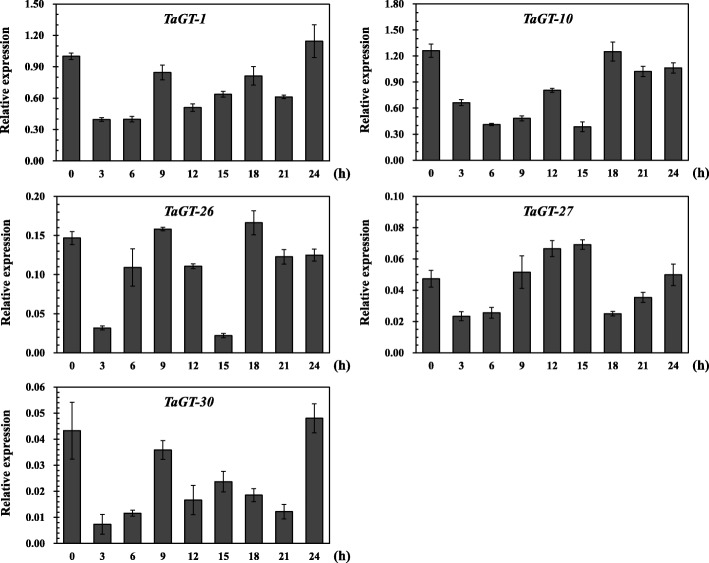
Expression patterns of wheat *trihelix* genes in response to photoperiod. Two-week-old seedling leaves were sampled at 0, 3, 6, 9, 12, 15, 18, 21, and 24 h under a 12-h light/12-h dark cycle. Bars reflect the means ± SD of three replicates

Evidence has shown that the transcription levels of some members belonging to the GT-2 clade decline in white light [36, 37]. To investigate the expression patterns of members in GT-2 clade in photoperiod sensing, qRT-PCR analyses were conducted. We analyzed the changes of the expression levels of *TaGT-1*, *TaGT-10*, *TaGT-26*, *TaGT-27*, and *TaGT-30* in two-week-old wheat seedling leaves under a 12 h light/12 h dark cycle (Fig. 5). The results showed that these five *trihelix* genes in GT-2 clade shared relatively similar expression profiles in response to photoperiod. The transcript levels of these genes began to decrease rapidly from 0 h and then gradually increased. These results are similar to those of previous studies. Except for *TaGT-27*, the transcript levels of the four other genes reached their first peaks at 9–12 h, then reached a second peak in the dark at 15–18 h, and then reached a third peak at approximately 24 h. The expression profile of *TaGT-27* was simpler because it only had two peaks (at 15 and 24 h). Differences in expression profiles between *TaGT-27* and the remaining four GT-2 clade genes may be related to their different motif compositions. *TaGT-27* has a motif composition similar to that of *Arabidopsis* GT-2 clade genes (*At5g28300* and *At5g47660*) and features a trihelix domain at the C-terminal, similar to all other members belonging to the GT-2 clade; however, this gene lacks the trihelix domain at the N-terminal, which *trihelix* genes usually have.

**Fig. 6:**
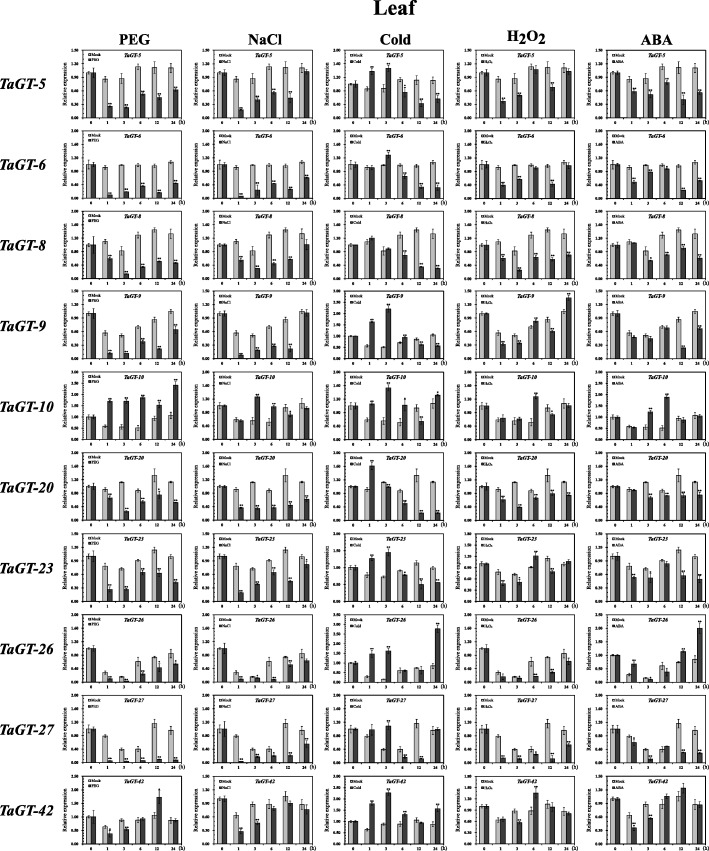
Expression profiles of wheat *trihelix* genes in seedling leaves under different treatments. Two-week-old seedling leaves were collected at 0, 1, 3, 6, 12, and 24 h under stress conditions of 20% (v/v) PEG6000, 200 mM NaCl, cold (4 °C), 10 mM H_2_O_2_, and 100 mM ABA. Three independent experiments were conducted. Error bars indicate SD. Statistically significant differences from the mock group are indicated as ^*^P < 0.05; ^**^P < 0.01

**Fig. 7:**
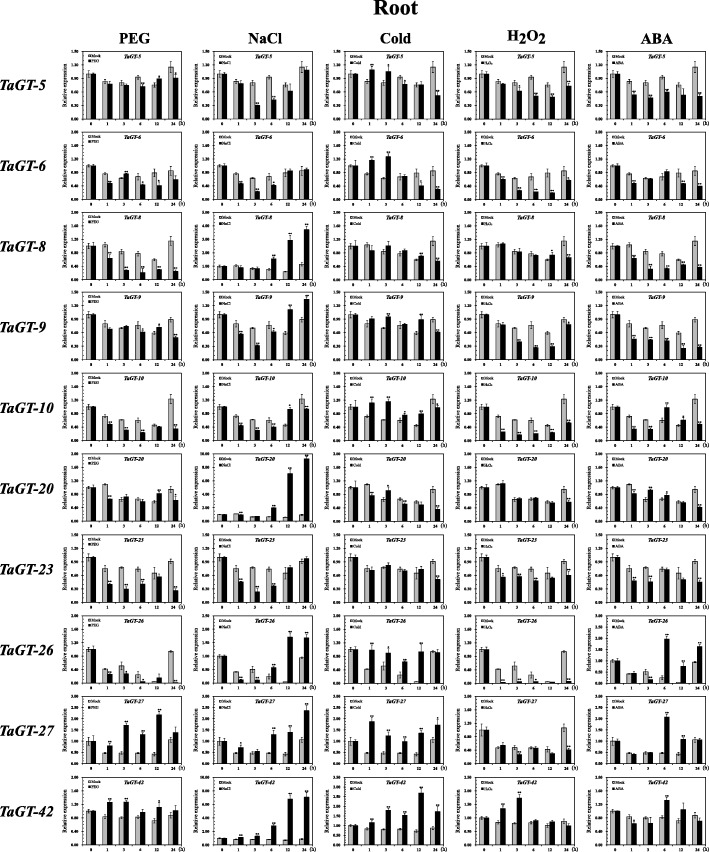
Expression profiles of wheat *trihelix* genes in seedling roots under different treatments. Two-week-old seedling roots were collected at 0, 1, 3, 6, 12, and 24 h under stress conditions of 20% (v/v) PEG6000, 200 mM NaCl, cold (4 °C), 10 mM H_2_O_2_, and 100 mM ABA. Three independent experiments were conducted. Error bars indicate SD. Statistically significant differences from the mock group are indicated as ^*^P < 0.05; ^**^P < 0.01

Recent studies indicate that *trihelix* genes play crucial roles in response to phytohormones and abiotic stresses. Using RNA-seq data based on IWGSC 1.1 genome annotations obtained from the expVIP website, differentially expressed wheat *trihelix* genes were analyzed under cold and drought stresses, and MA plots were drawn (Additional file 2: Figures S7–S9, Additional file 1: Table S6). Then, ten *trihelix* genes were selected to validate their responses against abiotic stresses further. We analyzed the transcript levels of these genes in the roots and leaves of two-week-old wheat seedlings under different abiotic stresses, including drought (PEG), salt (NaCl), cold (4 °C), and H_2_O_2_ stress treatments. Since the ABA signaling pathway is key in plant response to drought and salt stresses [29, 38], we also analyzed the expression profiles of these genes under exogenous ABA treatment (Figs. 6 and 7).

Based on the analysis results of RNA-seq data of leaves under cold stress, *TaGT-10*, *TaGT-26*, and *TaGT-27* were significantly up-regulated, and *TaGT-8* and *TaGT-20* were significantly down-regulated. The expression level of *ShCIGT* (homologous with *TaGT-42*) increased in tomato under cold and drought stresses [20]. *AtGT2L* is homologous with *TaGT-27* and up-regulated under cold, salt, and ABA treatments in *Arabidopsis* [17]. The expression profiles of the above wheat *trihelix* genes were confirmed in our qRT-PCR experiment. *TaGT-42* in roots was significantly up-regulated under cold stress. Interestingly, its expression level in roots showed no significant difference during the initial period of salt stress but was remarkably up-regulated by nearly 7-fold 12 h after salt treatment. The expression profiles of *TaGT-27* conformed to its homologous gene *AtGT2L* under cold, salt, and ABA treatments in roots. Furthermore, *TaGT-26* and *TaGT-27* showed very similar expression profiles under different treatments.

The results of RNA-seq data analysis of drought stress in leaves reveal that *TaGT-10* was significantly up-regulated, whereas *TaGT-6*, *TaGT-9*, *TaGT-26*, and *TaGT-27* were significantly down-regulated. The expression profiles of these five *trihelix* genes were validated by the results of qRT-PCR. *Arabidopsis GT-4*, which is homologous with *TaGT-9*, was reported up-regulated under salt stress [18]. Our qRT-PCR results showed that *TaGT-9* was down-regulated in roots to a certain extent at 0–6 h under salt stress but remarkably up-regulated after 6 h. The transcript level of *TaGT-9* was found to have remarkably increased in seedling leaves under cold stress.

*Trihelix* genes of GTγ clade in rice respond to diverse abiotic stresses, especially to salt stress [15]. qRT-PCR experiments were conducted to analyze the expression profiles of 3 GTγ clade genes (*TaGT-5*, *TaGT-20*, *TaGT-23*) under abiotic stresses and exogenous ABA treatments. *TaGT-5* and *TaGT-23* were down-regulated both in leaves and roots under salt stress, whereas *TaGT-20* was significantly up-regulated in roots. Furthermore, the results showed that all 3 genes responded to cold stress in seedling leaves.

## Discussion

As sessile organisms, wheat undergoes a variety of abiotic stresses, including salinity, drought, and extreme temperatures [29]. Wheat shares the same ancestor with *B. distachyon* and rice [39], and it is derived from two naturally interspecific hybridization events of three diploid donor species, two of which are the ancestors of *T. urartu* and *Ae. tauschii* [40, 41]. In our study, 94, 22, 29, and 31 *trihelix* genes were identified in *T. aestivum*, *T. urartu*, *Ae. tauschii*, and *B. distachyon*, respectively. The chromosomal distributions showed that no *trihelix* gene was located on wheat chromosome 7A, 7B, or 7D (Fig. 1); these results are consistent with the results of *T. urartu* and *Ae. tauschii* (no *trihelix* gene located on chromosome 7A of *T. urartu* or 7D of *Ae. tauschii*).

Due to the incompleteness of genome sequencing results, some *trihelix* gene family members probably are not identified in *T. urartu* or *Ae. tauschii*. The average number of *trihelix* genes on each subgenome of wheat is 31, which is similar to the previous studies in *Arabidopsis* (30) and rice (31). Ninety-four *trihelix* genes were clustered in 5 clades. The members belonging to the same clades usually share similar motif compositions and exon/intron structures, and they may have similar functions (Fig. 3). Our results showed the conservation in wheat *trihelix* gene evolution. Interspecific comparisons among the genomes of grass plants revealed more intrachromosomal gene duplication events in the wheat genome than in the genomes of other grass species [33]. In our study, five pairs of tandem duplication genes were identified in the wheat *trihelix* gene family, and only one gene corresponding to that presented in the equivalent genomic regions of rice and *B. distachyon*. Tandem duplication events occurred twice among *TaGT-12*, *TaGT-13*, and *TaGT-14*, thereby formed a cluster of three tandem duplication genes. Thus, gene duplication events are the main driving force for the *trihelix* gene evolution during the speciation and evolution of bread wheat.

The tissue-specific expression of genes usually reflects their corresponding functions. Our results indicated that the tandem duplication genes *TaGT-12*, *TaGT-13,* and TaGT-14 show nearly no expression in all tested wheat tissues (Fig. 4, Additional file 1: Table S5). The homologous genes *TaGT-44*/*TaGT-77* and *TaGT-45*/*TaGT-78* had similar expression profiles but were specifically expressed in grains, thereby showing that their functions may be associated with grain development. Further expression profile analysis of wheat *trihelix* homologous genes revealed that three homologous genes on subgenomes A, B, and D often have similar expression characteristics but different expression levels. Generally, homologous *trihelix* genes on subgenomes A and B, or subgenomes A and D have the same expression levels, and the rest one had relatively higher or lower expression level. For instance, *TaGT-5*, *TaGT-36*, and *TaGT-68* are homologous genes, and they have similar expression characteristics. *TaGT-5* and *TaGT-68* belong to group I, and their transcript levels were very similar in all the tissues. By contrast, TaGT-36 belongs to group III, which showed approximately twice the expression levels of *TaGT-5* and *TaGT-68* in all tested tissues.

In this study, we selected ten wheat *trihelix* genes to analyze their expression profiles under different stress treatments, including PEG, NaCl, cold, H_2_O_2_, and exogenous ABA (Figs. 6 and 7). The results of expression analysis of selected wheat *trihelix* genes were essentially consistent with the results of RNA-seq data and published literatures. *TaGT-10* was reported to be significantly up-regulated in leaves under PEG stress for 12 h [19]. Our qRT-PCR results showed that the transcript level of *TaGT-10* reached the first peak under PEG stress at 12 h and was up-regulated again 2.4-fold at 24 h compared with the mock group. *TaGT-10* revealed different expression profiles in leaves and roots under PEG stress. The transcript level of *TaGT-10* in roots constantly decreased within 24 h under PEG stress. *TaGT-27* is homologous with *AtGT2L*, which was reported to be up-regulated under salt, cold, and ABA stresses [17]. The similar result of *TaGT-27* was validated via qRT-PCR in our research. In addition, *TaGT-27* was found to be up-regulated in roots under PEG stress. *OsGTγ-1* (*Os02g33770*), *OsGTγ-2* (*Os11g06410*), and *OsGTγ-3* (*Os12g06640*) were significantly up-regulated in rice under salt stress, but their expression data were only analyzed at 0–6 h [15]. We found that the GTγ clade genes *TaGT-5* and *TaGT-23* were down-regulated under salt stress, whereas *TaGT-20* (homologous with *OsGTγ-2*) was significantly up-regulated in roots. *TaGT-20* and *OsGTγ-2* had similar expression profiles under salt stress, exhibited no significant change at 0–3 h and started to be significantly up-regulated at 6 h. Further findings indicated that the expression levels of *TaGT-20* were 7- and 9-fold those of the mock group at 12 and 24 h. *ShCIGT* responded to cold and drought stresses, and *AtGT-3b* responded to salt stress [20, 42]. Both of these genes are homologous with *TaGT-42*. The expression level of *TaGT-42* increased in roots under cold, PEG, and NaCl stresses in our study; it was also increased under H_2_O_2_ stress. The expression profile of *TaGT-42* in roots under NaCl stress was similar to those of *TaGT-8*, *TaGT-20*, *TaGT-26*, and *TaGT-27*. These genes showed nearly no change at the initial stage of stress and began to be up-regulated at approximately 6 h. *TaGT-42* was continually up-regulated by more than 7-fold at 12 and 24 h. The result indicated that *TaGT-42* and *TaGT-20* may indirectly participate in response to salt stress and directly respond to secondary stresses, such as oxidative damage.

## Conclusions

In summary, our study is the first genome-wide analysis of *trihelix* genes in wheat and its close relatives. By integrating ortholog identification, phylogenetic analysis, tandem and segmental duplication identification, and conserved motif and structural analysis, comparative analysis with the available genomic information of wheat and its relatives was conducted to enable exploration of the evolution process of the identified *trihelix* genes. Some *trihelix* genes were confirmed to participate in response to multiple abiotic stresses, based on the tissue-specific expression patterns and the results of differential expression analysis under abiotic stresses. The results of our study build the foundation for stress-resistant breeding of wheat and its relatives.

## Methods

### Identification and characterization of *trihelix* genes

The wheat genome and protein database (IWGSC RefSeq v1.0, hexaploid bread wheat variety Chinese Spring) was downloaded from URGI (https://wheat-urgi.versailles.inra.fr/Seq-Repository/Assemblies) [43]. The genome and protein databases of *T. urartu*, *Ae. tauschii*, and *B. distachyon* were downloaded from MBKbase (http://www.mbkbase.org/Tu/), Sequencing the *Aegilops tauschii* Genome (http://aegilops.wheat.ucdavis.edu/ATGSP/annotation/), and JGI Phytozome 12 (http://phytozome.jgi.doe.gov/), respectively [39–41]. The HMM profile (PF13837) was downloaded from Pfam (http://pfam.xfam.org/family/pf13837) and used to search trihelix domains through HMMER3.0 software (http://hmmer.org/download.html) [44, 45]. The Python script was used to eliminate redundant sequences, and the sequence with the longest length was chosen as the representative of each gene. The SMART (http://smart.embl-heidelberg.de) and HMMER (https://www.ebi.ac.uk/Tools/hmmer/) websites were used to confirm the trihelix domain in all the identified *trihelix* genes [46, 47].

The theoretical pI and Mw of the genes were analyzed by uploading protein sequences to ExPASy (Compute pI/Mw tool, https://web.expasy.org/compute_pi/) [48]. WOLF PSORT (https://wolfpsort.hgc.jp/) was utilized to predict subcellular localization [49].

### Identification of orthologs

OrthoGNC software was used to precisely predict the pairwise orthology relations among *T. aestivum*, *T. urartu*, *Ae. tauschii*, *B. distachyon*, and *Oryza sativa* following the gene neighborhood conservation method [50].

### Phylogenetic analysis and gene synteny analysis

MEGA7.0 was utilized to align the full-length sequences of trihelix proteins in wheat, *B. distachyon*, rice, and *Arabidopsis*, and we used the neighbor-joining method with a bootstrap value of 1000 replicates and default parameters to construct the unrooted phylogenetic tree [51]. To analyze synteny relationships among the *T. aestivum* and *B. distachyon* and *O. sativa* genomes, MCScanX was used with default settings [52, 53], and the figures were drawn using Circos 0.69 [54].

### Motif and structural analysis

To analyze the conservative motifs of trihelix TFs, the MEME tool (http://meme-suite.org/tools/meme) was used [55]. The limits of the maximum number of motifs are specified as 13. To visualize the exon/intron structures of *trihelix* gene family members, coding sequences (CDS) and genomic sequences were uploaded to Gene Structure Display Server 2.0 (http://gsds.cbi.pku.edu.cn/) [56].

### Expression profile analysis

To analyze tissue-specific expression patterns of *trihelix* genes in wheat, we downloaded the RNA-seq data from the expVIP website (http://www.wheat-expression.com/) [57, 58]. The study title was “choulet_URGI”, and 15 types of tissues from hexaploid wheat were involved. Gplots package of R program (https://www.R-project.org/) was used to draw the heatmap.

We also downloaded the RNA-seq data titled “SRP043554” and “SRP045409” to analyze the expression profiles of wheat *trihelix* genes under cold and drought stresses. Using the DESeq2 package of R, we analyzed the differential expression of wheat *trihelix* genes and generated the MA plots [59]. Points highlighted in red represent significantly differentially expressed *trihelix* genes (padj < 0.05). Open triangles pointing either up or down indicate the points falling out of the window.

### Plant materials

Seeds of *T. aestivum* Chinese Spring were stored in our laboratory. The seeds were germinated in distilled water under the dark condition and cultured in the greenhouse with a 12-h light/12-h dark cycle at 22 °C. To conduct the qRT-PCR analysis of wheat *trihelix* genes in response to photoperiod, the leaves of two-week-old wheat seedlings were sampled every 3 h for nine continuous time points (0, 3, 6, 9, 12, 15, 18, 21 and 24 h). To enable the expression profile analysis of wheat *trihelix* genes under abiotic stresses, leaves and roots of two-week-old seedlings were sampled at 0, 1, 3, 6, 12, and 24 h after treatment with 20% (v/v) PEG6000, 200 mM NaCl, 10 mM H_2_O_2_, 100 mM ABA, and cold condition (4 °C). All of the samples were collected and stored at − 80 °C. The experiments were conducted with three independent biological replicates.

### Total RNA isolation and qRT-PCR analysis

A total RNA extraction kit (Zomanbio, China) was used to extract total RNA from wheat. A PrimeScript™ RT reagent kit with gDNA Eraser (Perfect Real Time; Takara, Japan) was used to synthesize the first cDNA chain. qPCR SYBR Green Mix (Vazyme, China) was used to conduct qRT-PCR analysis in a CFX™ real-time PCR detection system (Bio-Rad, USA). The primer sequences used in this study are indicated in Additional file 1: Table S7. *TaActin* (GenBank ID: AB181991.1) was used as the internal control. Three technical replicates were used to analyze each sample, and the expression data were analyzed via the 2^-ΔΔCT^ method [60].

## Additional files

Additional file 1: **Table S1.** Characteristic features of *trihelix* genes in wheat and its relatives. **Table S2.** Coding sequences of *trihelix* genes in wheat and its relatives. **Table S3.** Amino acid sequences of trihelix proteins in wheat and its relatives. **Table S4.** Syntenic relationships of *trihelix* genes between wheat and its relatives. **Table S5.** Expression data of wheat *trihelix* genes for tissue-specific expression profile analyses. **Table S6.** Differential expression of wheat *trihelix* genes under cold and drought stresses. **Table S7.** Primers for qRT-PCR.

Additional file 2: **Figure S1.** Locations and duplication events of *trihelix* genes on *T. urartu* chromosomes. Red boxes indicate tandem duplications. **Figure S2.** Locations and duplication events of *trihelix* genes on *Ae. tauschii* chromosomes. Red boxes indicate tandem duplications, and red lines indicate segmental duplications. **Figure S3.** Locations and duplication events of *trihelix* genes on *B. distachyon* chromosomes. Red lines indicate segmental duplications. **Figure S4.** Syntenic analysis of *trihelix* genes between wheat and rice. Red, blue, and green bands represent subgenomes A, B, and D, respectively. Yellow bands indicate the rice genome. **Figure S5.** Syntenic analysis of *trihelix* genes between wheat and *B. distachyon*. Red, blue, and green bands represent subgenomes A, B, and D, respectively. Yellow bands indicate the rice genome. **Figure S6.** Conserved motifs of trihelix proteins. The logos of the motifs were predicted using MEME. **Figure S7.** MA plots of the differential expression of wheat *trihelix* genes under cold stress. **Figure S8.** MA plots of the differential expression of wheat *trihelix* genes under drought stress for 1 h. **Figure S9.** MA plots of the differential expression of wheat *trihelix* genes under drought stress for 6 h.

## Abbreviations

FAO: Food and Agriculture Organization
HMM: Hidden Markov Model
IWGSC: The International Wheat Genome Sequencing Consortium
MW: Molecular weight
padj: adjusted *p* value
pI: Isoelectric point
TF: Transcription factor
TPM: transcript per million
