# Supplementary figures and images for: Genome-wide identification and expression profiling of trihelix gene family under abiotic stresses in wheat
Source: BMC Genomics. 2019 Apr 11;20:287. doi: 10.1186/s12864-019-5632-2 (PMC6460849; doi:10.1186/s12864-019-5632-2)

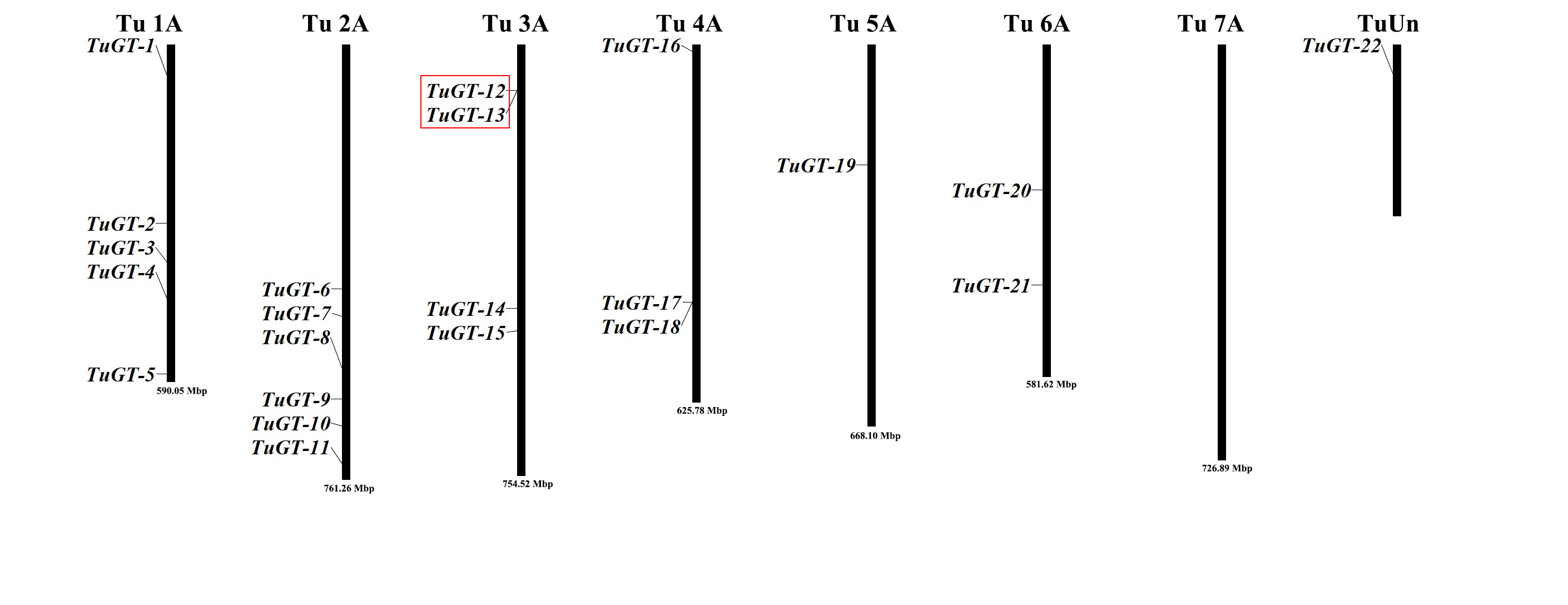

Supplement: Supplementary file 2 — Figure S1. Locations and duplication events of trihelix genes on T. urartu chromosomes. Red boxes indicate tandem duplications. Figure S2. Locations and duplication events of trihelix genes on Ae. tauschii chromosomes. Red boxes indicate tandem duplications, and red lines indicate segmental duplications. Figure S3. Locations and duplication events of trihelix genes on B. distachyon chromosomes. Red lines indicate segmental duplications. Figure S4. Syntenic analysis of trihelix genes between wheat and rice. Red, blue, and green bands represent subgenomes A, B, and D, respectively. Yellow bands indicate the rice genome. Figure S5. Syntenic analysis of trihelix genes between wheat and B. distachyon. Red, blue, and green bands represent subgenomes A, B, and D, respectively. Yellow bands indicate the rice genome. Figure S6. Conserved motifs of trihelix proteins. The logos of the motifs were predicted using MEME. Figure S7. MA plots of the differential expression of wheat trihelix genes under cold stress. Figure S8. MA plots of the differential expression of wheat trihelix genes under drought stress for 1 h. Figure S9. MA plots of the differential expression of wheat trihelix genes under drought stress for 6 h. [file 12864_2019_5632_MOESM2_ESM.zip › Additional file 2 Figure S1.jpg]

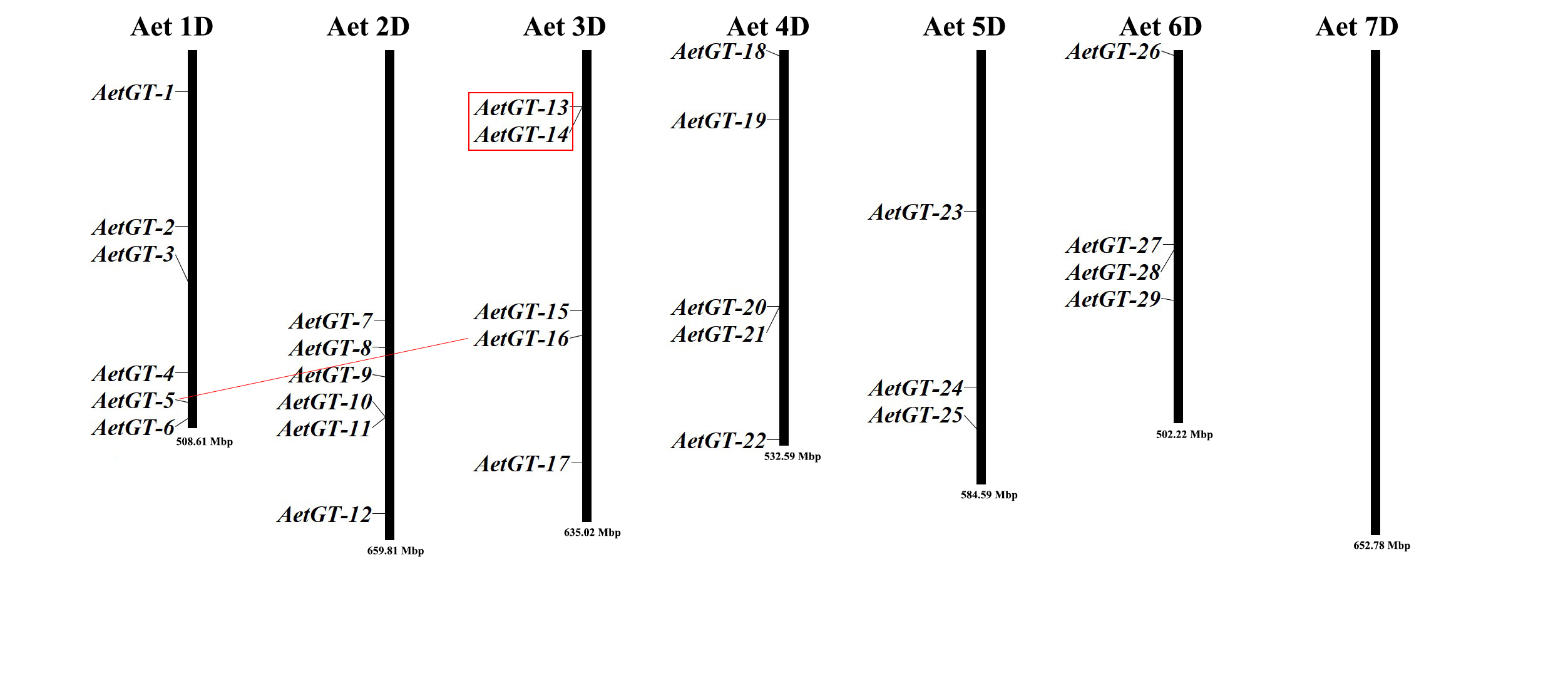

Supplement: Supplementary file 2 — Figure S1. Locations and duplication events of trihelix genes on T. urartu chromosomes. Red boxes indicate tandem duplications. Figure S2. Locations and duplication events of trihelix genes on Ae. tauschii chromosomes. Red boxes indicate tandem duplications, and red lines indicate segmental duplications. Figure S3. Locations and duplication events of trihelix genes on B. distachyon chromosomes. Red lines indicate segmental duplications. Figure S4. Syntenic analysis of trihelix genes between wheat and rice. Red, blue, and green bands represent subgenomes A, B, and D, respectively. Yellow bands indicate the rice genome. Figure S5. Syntenic analysis of trihelix genes between wheat and B. distachyon. Red, blue, and green bands represent subgenomes A, B, and D, respectively. Yellow bands indicate the rice genome. Figure S6. Conserved motifs of trihelix proteins. The logos of the motifs were predicted using MEME. Figure S7. MA plots of the differential expression of wheat trihelix genes under cold stress. Figure S8. MA plots of the differential expression of wheat trihelix genes under drought stress for 1 h. Figure S9. MA plots of the differential expression of wheat trihelix genes under drought stress for 6 h. [file 12864_2019_5632_MOESM2_ESM.zip › Additional file 2 Figure S2.jpg]

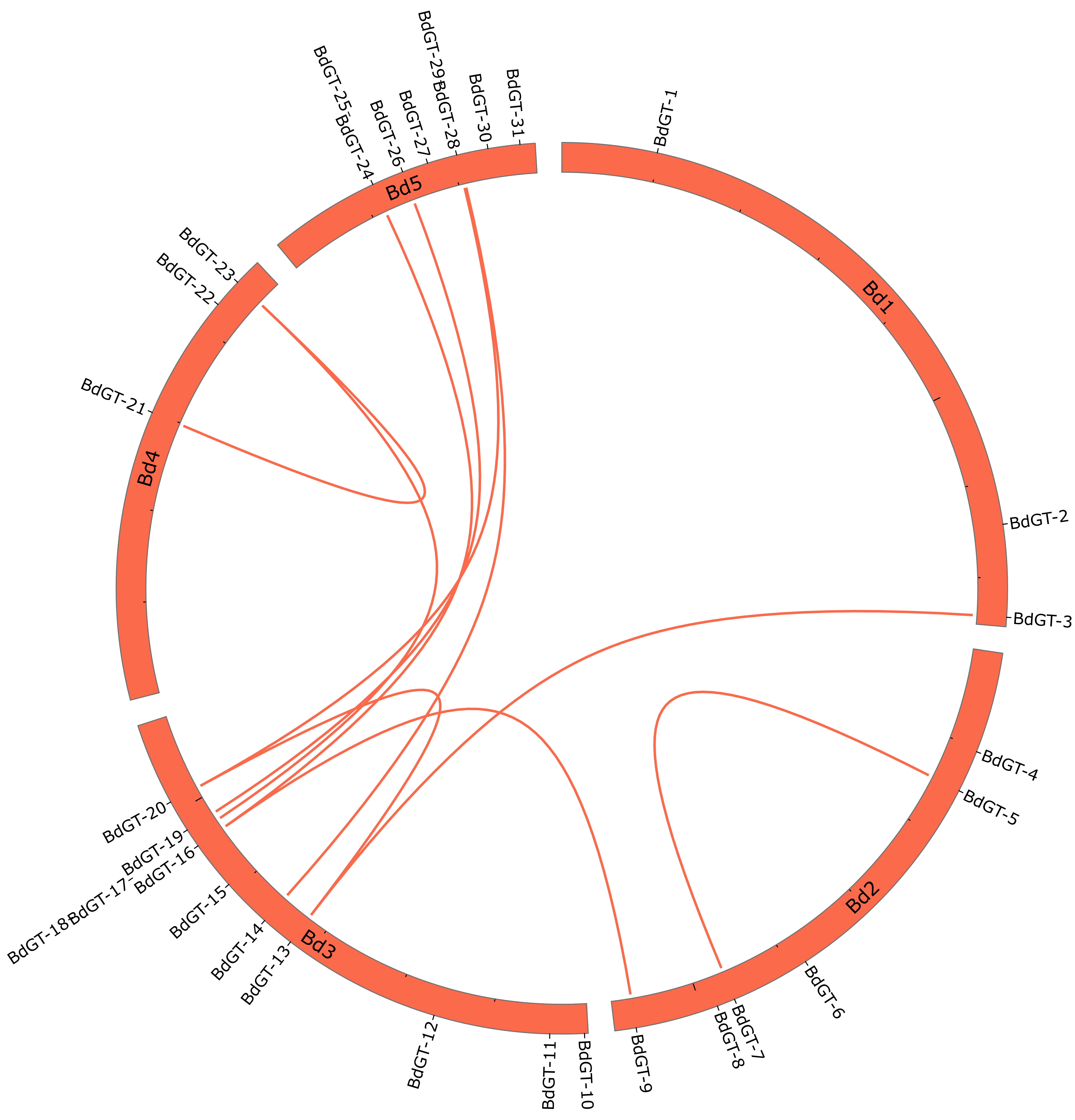

Supplement: Supplementary file 2 — Figure S1. Locations and duplication events of trihelix genes on T. urartu chromosomes. Red boxes indicate tandem duplications. Figure S2. Locations and duplication events of trihelix genes on Ae. tauschii chromosomes. Red boxes indicate tandem duplications, and red lines indicate segmental duplications. Figure S3. Locations and duplication events of trihelix genes on B. distachyon chromosomes. Red lines indicate segmental duplications. Figure S4. Syntenic analysis of trihelix genes between wheat and rice. Red, blue, and green bands represent subgenomes A, B, and D, respectively. Yellow bands indicate the rice genome. Figure S5. Syntenic analysis of trihelix genes between wheat and B. distachyon. Red, blue, and green bands represent subgenomes A, B, and D, respectively. Yellow bands indicate the rice genome. Figure S6. Conserved motifs of trihelix proteins. The logos of the motifs were predicted using MEME. Figure S7. MA plots of the differential expression of wheat trihelix genes under cold stress. Figure S8. MA plots of the differential expression of wheat trihelix genes under drought stress for 1 h. Figure S9. MA plots of the differential expression of wheat trihelix genes under drought stress for 6 h. [file 12864_2019_5632_MOESM2_ESM.zip › Additional file 2 Figure S3.pdf]

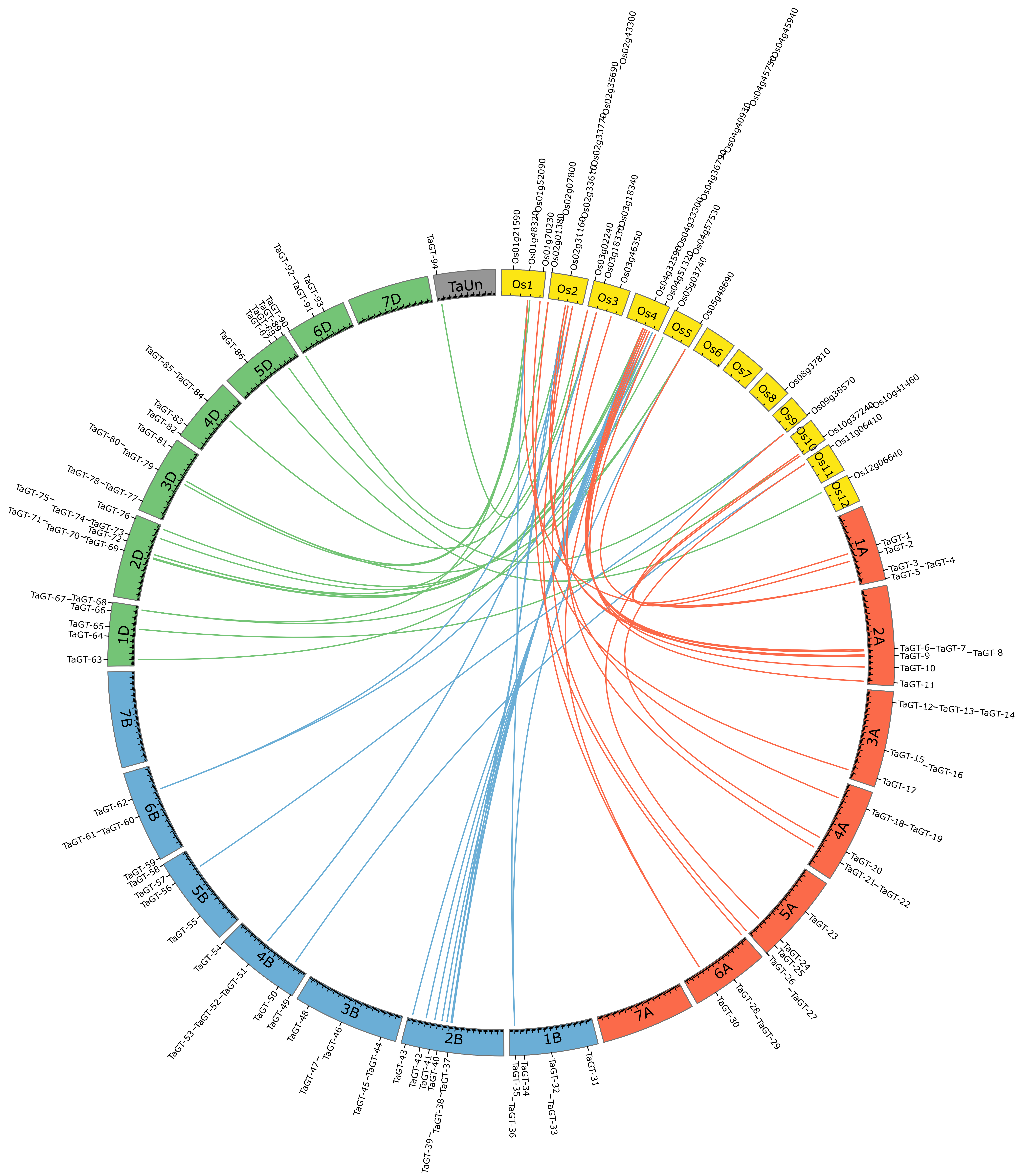

Supplement: Supplementary file 2 — Figure S1. Locations and duplication events of trihelix genes on T. urartu chromosomes. Red boxes indicate tandem duplications. Figure S2. Locations and duplication events of trihelix genes on Ae. tauschii chromosomes. Red boxes indicate tandem duplications, and red lines indicate segmental duplications. Figure S3. Locations and duplication events of trihelix genes on B. distachyon chromosomes. Red lines indicate segmental duplications. Figure S4. Syntenic analysis of trihelix genes between wheat and rice. Red, blue, and green bands represent subgenomes A, B, and D, respectively. Yellow bands indicate the rice genome. Figure S5. Syntenic analysis of trihelix genes between wheat and B. distachyon. Red, blue, and green bands represent subgenomes A, B, and D, respectively. Yellow bands indicate the rice genome. Figure S6. Conserved motifs of trihelix proteins. The logos of the motifs were predicted using MEME. Figure S7. MA plots of the differential expression of wheat trihelix genes under cold stress. Figure S8. MA plots of the differential expression of wheat trihelix genes under drought stress for 1 h. Figure S9. MA plots of the differential expression of wheat trihelix genes under drought stress for 6 h. [file 12864_2019_5632_MOESM2_ESM.zip › Additional file 2 Figure S4.pdf]

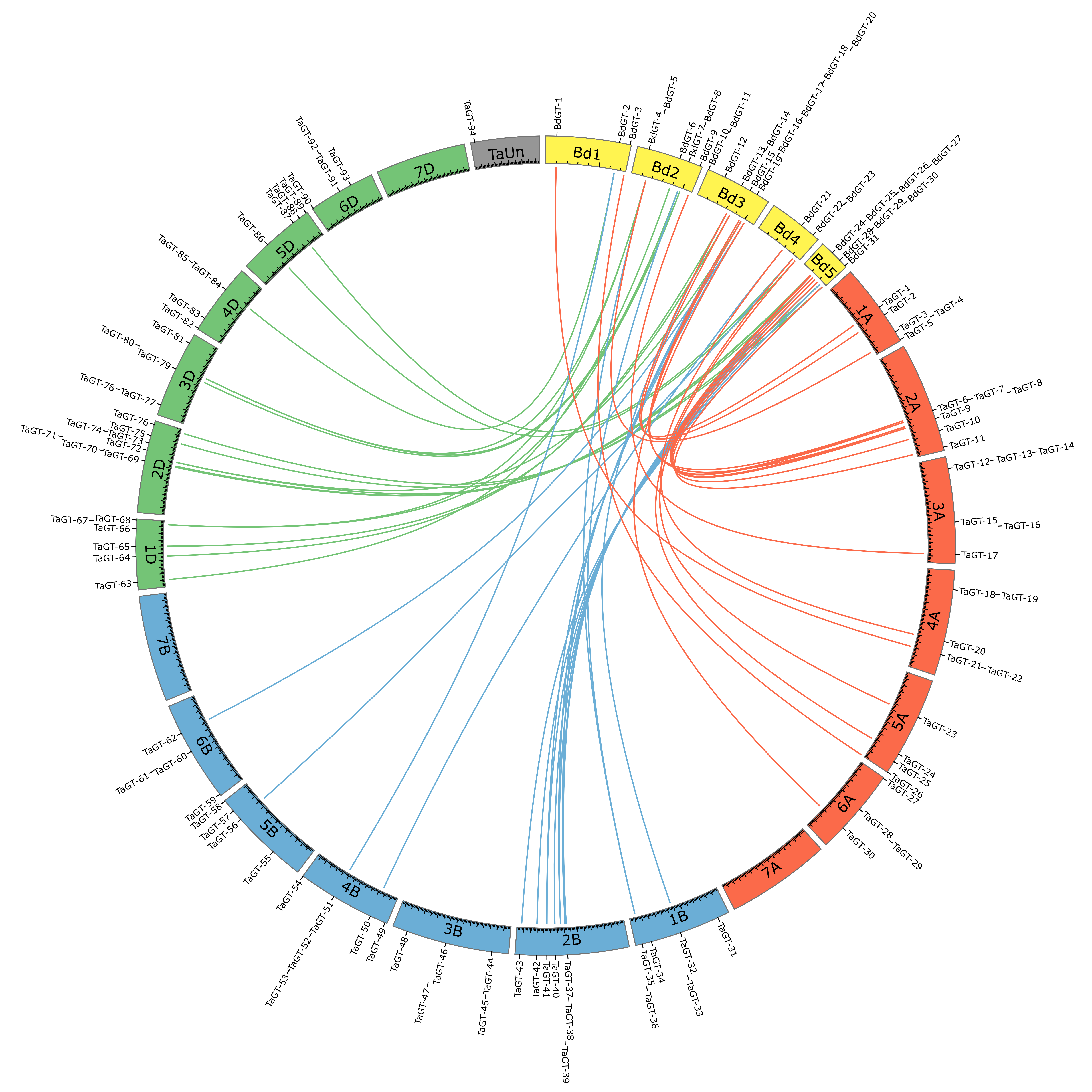

Supplement: Supplementary file 2 — Figure S1. Locations and duplication events of trihelix genes on T. urartu chromosomes. Red boxes indicate tandem duplications. Figure S2. Locations and duplication events of trihelix genes on Ae. tauschii chromosomes. Red boxes indicate tandem duplications, and red lines indicate segmental duplications. Figure S3. Locations and duplication events of trihelix genes on B. distachyon chromosomes. Red lines indicate segmental duplications. Figure S4. Syntenic analysis of trihelix genes between wheat and rice. Red, blue, and green bands represent subgenomes A, B, and D, respectively. Yellow bands indicate the rice genome. Figure S5. Syntenic analysis of trihelix genes between wheat and B. distachyon. Red, blue, and green bands represent subgenomes A, B, and D, respectively. Yellow bands indicate the rice genome. Figure S6. Conserved motifs of trihelix proteins. The logos of the motifs were predicted using MEME. Figure S7. MA plots of the differential expression of wheat trihelix genes under cold stress. Figure S8. MA plots of the differential expression of wheat trihelix genes under drought stress for 1 h. Figure S9. MA plots of the differential expression of wheat trihelix genes under drought stress for 6 h. [file 12864_2019_5632_MOESM2_ESM.zip › Additional file 2 Figure S5.pdf]

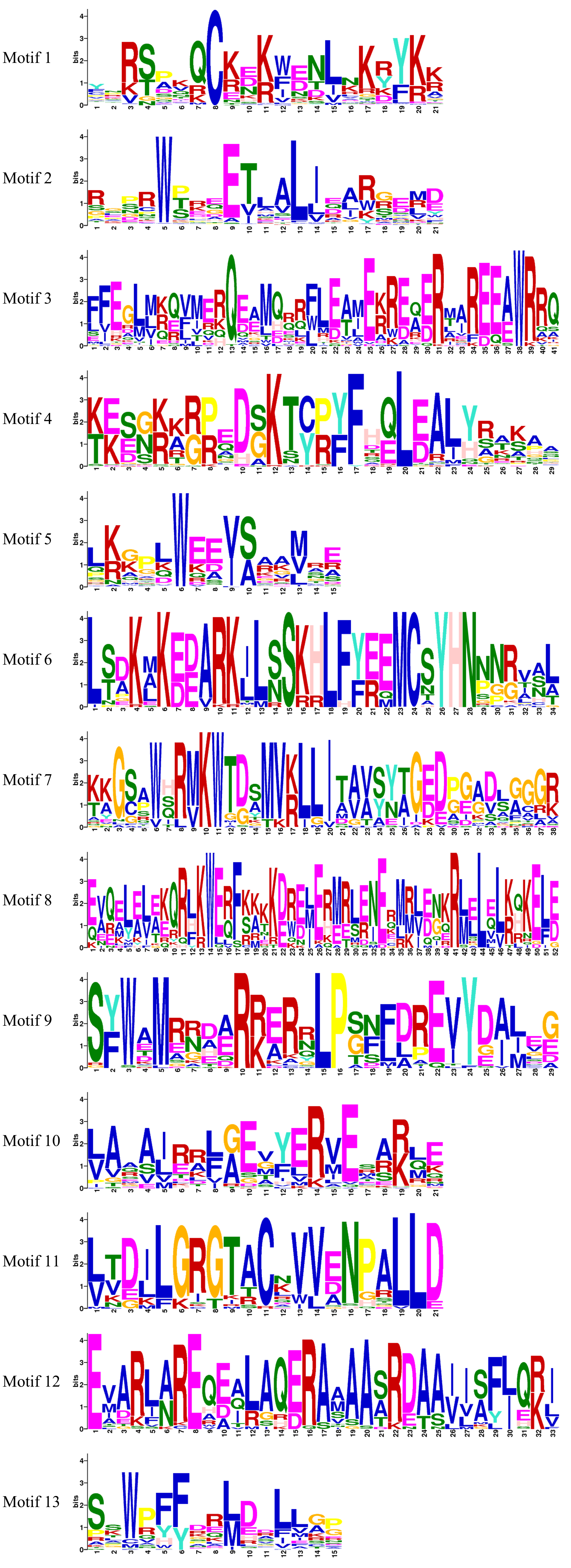

Supplement: Supplementary file 2 — Figure S1. Locations and duplication events of trihelix genes on T. urartu chromosomes. Red boxes indicate tandem duplications. Figure S2. Locations and duplication events of trihelix genes on Ae. tauschii chromosomes. Red boxes indicate tandem duplications, and red lines indicate segmental duplications. Figure S3. Locations and duplication events of trihelix genes on B. distachyon chromosomes. Red lines indicate segmental duplications. Figure S4. Syntenic analysis of trihelix genes between wheat and rice. Red, blue, and green bands represent subgenomes A, B, and D, respectively. Yellow bands indicate the rice genome. Figure S5. Syntenic analysis of trihelix genes between wheat and B. distachyon. Red, blue, and green bands represent subgenomes A, B, and D, respectively. Yellow bands indicate the rice genome. Figure S6. Conserved motifs of trihelix proteins. The logos of the motifs were predicted using MEME. Figure S7. MA plots of the differential expression of wheat trihelix genes under cold stress. Figure S8. MA plots of the differential expression of wheat trihelix genes under drought stress for 1 h. Figure S9. MA plots of the differential expression of wheat trihelix genes under drought stress for 6 h. [file 12864_2019_5632_MOESM2_ESM.zip › Additional file 2 Figure S6.jpg]

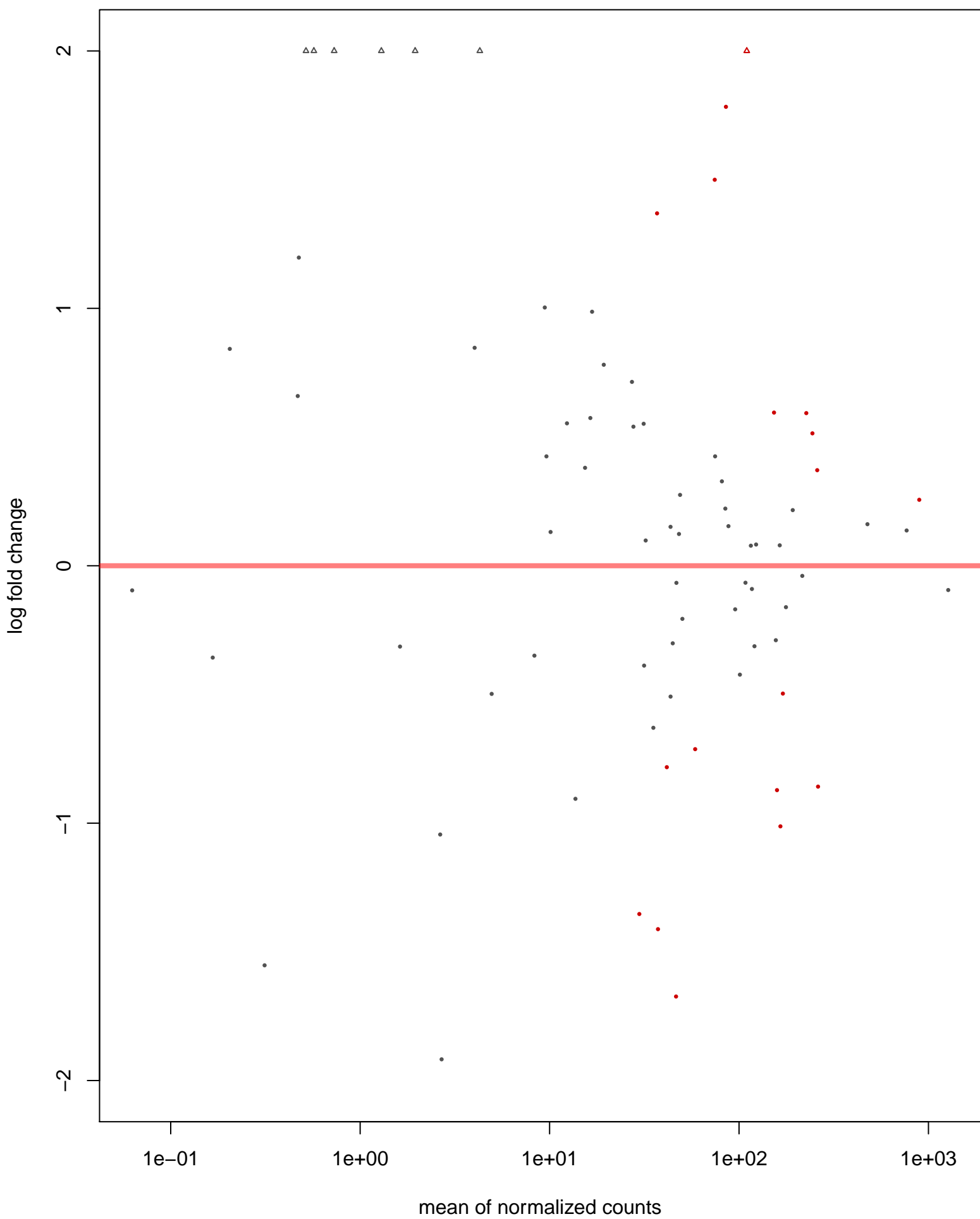

Supplement: Supplementary file 2 — Figure S1. Locations and duplication events of trihelix genes on T. urartu chromosomes. Red boxes indicate tandem duplications. Figure S2. Locations and duplication events of trihelix genes on Ae. tauschii chromosomes. Red boxes indicate tandem duplications, and red lines indicate segmental duplications. Figure S3. Locations and duplication events of trihelix genes on B. distachyon chromosomes. Red lines indicate segmental duplications. Figure S4. Syntenic analysis of trihelix genes between wheat and rice. Red, blue, and green bands represent subgenomes A, B, and D, respectively. Yellow bands indicate the rice genome. Figure S5. Syntenic analysis of trihelix genes between wheat and B. distachyon. Red, blue, and green bands represent subgenomes A, B, and D, respectively. Yellow bands indicate the rice genome. Figure S6. Conserved motifs of trihelix proteins. The logos of the motifs were predicted using MEME. Figure S7. MA plots of the differential expression of wheat trihelix genes under cold stress. Figure S8. MA plots of the differential expression of wheat trihelix genes under drought stress for 1 h. Figure S9. MA plots of the differential expression of wheat trihelix genes under drought stress for 6 h. [file 12864_2019_5632_MOESM2_ESM.zip › Additional file 2 Figure S7.pdf]

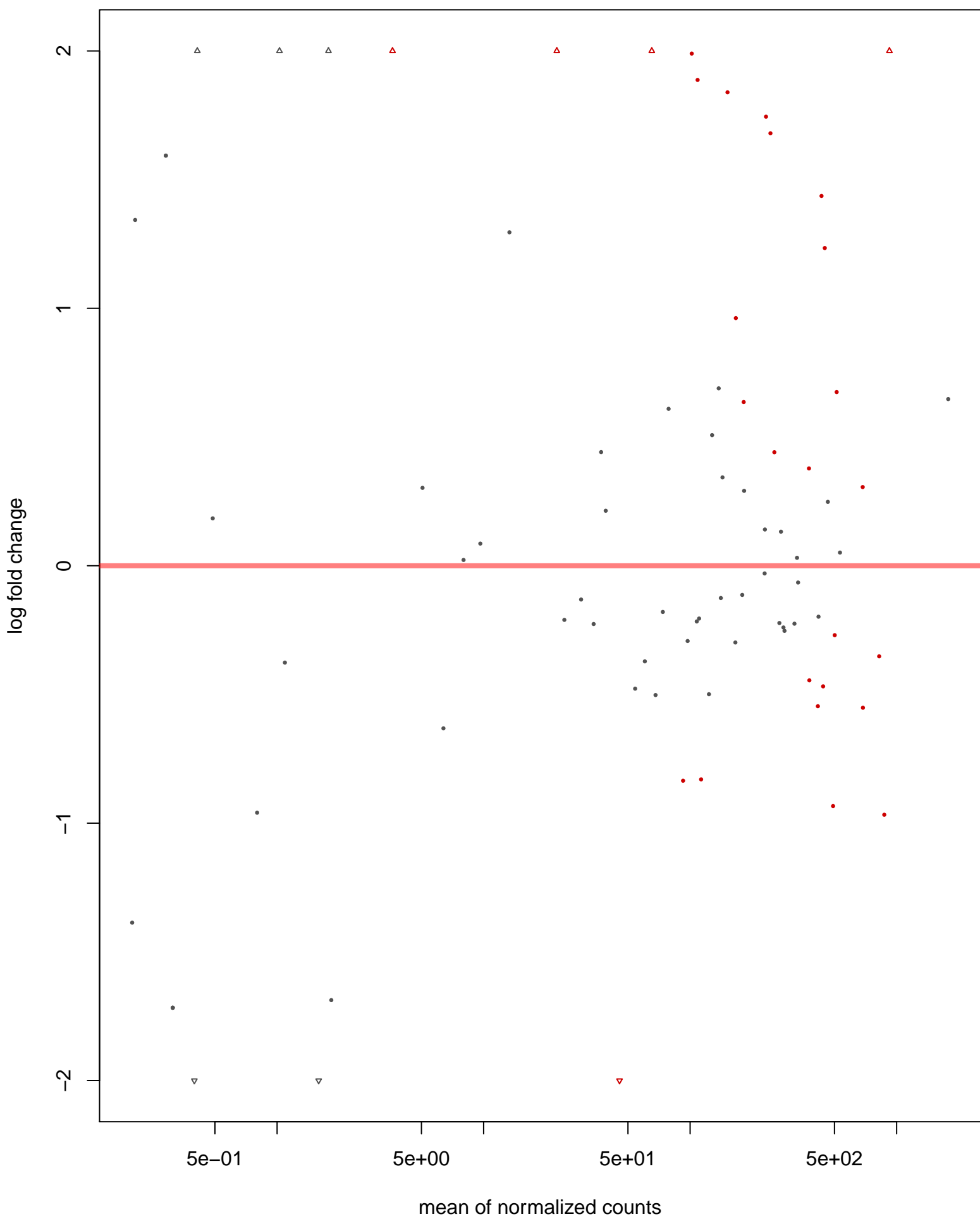

Supplement: Supplementary file 2 — Figure S1. Locations and duplication events of trihelix genes on T. urartu chromosomes. Red boxes indicate tandem duplications. Figure S2. Locations and duplication events of trihelix genes on Ae. tauschii chromosomes. Red boxes indicate tandem duplications, and red lines indicate segmental duplications. Figure S3. Locations and duplication events of trihelix genes on B. distachyon chromosomes. Red lines indicate segmental duplications. Figure S4. Syntenic analysis of trihelix genes between wheat and rice. Red, blue, and green bands represent subgenomes A, B, and D, respectively. Yellow bands indicate the rice genome. Figure S5. Syntenic analysis of trihelix genes between wheat and B. distachyon. Red, blue, and green bands represent subgenomes A, B, and D, respectively. Yellow bands indicate the rice genome. Figure S6. Conserved motifs of trihelix proteins. The logos of the motifs were predicted using MEME. Figure S7. MA plots of the differential expression of wheat trihelix genes under cold stress. Figure S8. MA plots of the differential expression of wheat trihelix genes under drought stress for 1 h. Figure S9. MA plots of the differential expression of wheat trihelix genes under drought stress for 6 h. [file 12864_2019_5632_MOESM2_ESM.zip › Additional file 2 Figure S8.pdf]

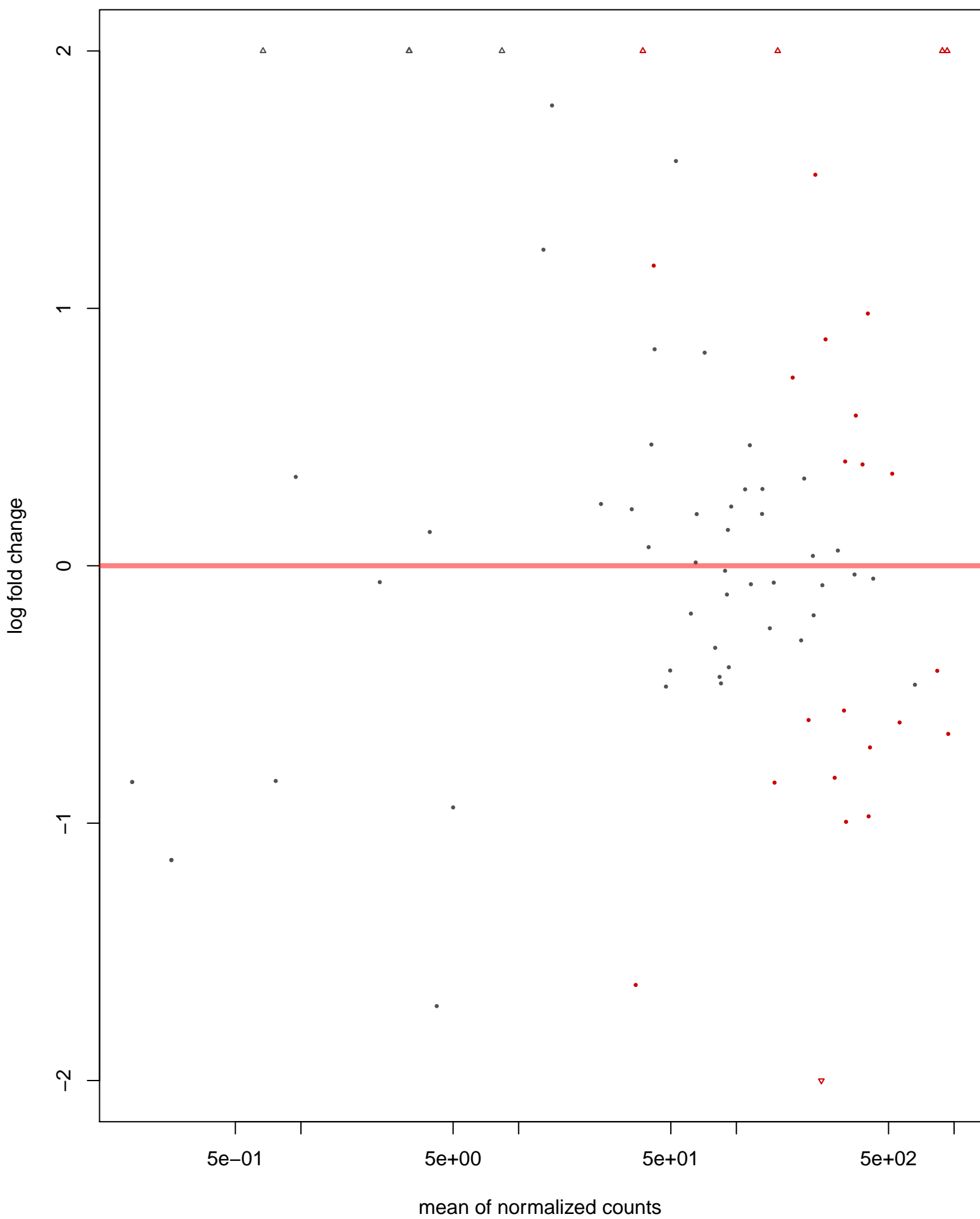

Supplement: Supplementary file 2 — Figure S1. Locations and duplication events of trihelix genes on T. urartu chromosomes. Red boxes indicate tandem duplications. Figure S2. Locations and duplication events of trihelix genes on Ae. tauschii chromosomes. Red boxes indicate tandem duplications, and red lines indicate segmental duplications. Figure S3. Locations and duplication events of trihelix genes on B. distachyon chromosomes. Red lines indicate segmental duplications. Figure S4. Syntenic analysis of trihelix genes between wheat and rice. Red, blue, and green bands represent subgenomes A, B, and D, respectively. Yellow bands indicate the rice genome. Figure S5. Syntenic analysis of trihelix genes between wheat and B. distachyon. Red, blue, and green bands represent subgenomes A, B, and D, respectively. Yellow bands indicate the rice genome. Figure S6. Conserved motifs of trihelix proteins. The logos of the motifs were predicted using MEME. Figure S7. MA plots of the differential expression of wheat trihelix genes under cold stress. Figure S8. MA plots of the differential expression of wheat trihelix genes under drought stress for 1 h. Figure S9. MA plots of the differential expression of wheat trihelix genes under drought stress for 6 h. [file 12864_2019_5632_MOESM2_ESM.zip › Additional file 2 Figure S9.pdf]
